# Fibroblast Growth Factor-2 facilitates the growth and chemo-resistance of leukemia cells in the bone marrow by modulating osteoblast functions

**DOI:** 10.1038/srep30779

**Published:** 2016-08-02

**Authors:** Keiki Sugimoto, Yasuhiko Miyata, Takayuki Nakayama, Shigeki Saito, Ritsuro Suzuki, Fumihiko Hayakawa, Satoshi Nishiwaki, Hiroki Mizuno, Kyosuke Takeshita, Hidefumi Kato, Ryuzo Ueda, Akiyoshi Takami, Tomoki Naoe

**Affiliations:** 1 Fujii Memorial Research Institute, Otsuka Pharmaceutical Co. Ltd., Otsu, Shiga, Japan; 2 Departments of Hematology, Nagoya Medical Center, Nagoya, Aichi, Japan; 3 Department of Transfusion Medicine, Aichi Medical University, Nagakute, Aichi, Japan; 4 Departments of Hematology, Japanese Red Cross Nagoya Daiini Hospital, Nagoya, Aichi, Japan; 5 Departments of Hematology, Shimane University, Izumo, Shimane, Japan; 6 Departments of Hematology, Nagoya University Graduate School of Medicine, Nagoya, Aichi, Japan; 7 Depertment of Hematology and Oncology, Toyohashi Municipal Hospital, Toyohashi, Aichi, Japan; 8 Laboratory of Cellular Dynamics, World Premier International Research Center Initiative-Immunology Frontier Research Center, Osaka University, Suita, Japan; 9 Departments of Cardiology, Nagoya University Graduate School of Medicine, Nagoya, Aichi, Japan; 10 Department of Tumor Immunology, Aichi Medical University, Nagakute, Aichi, Japan; 11 Department of Hematology, Aichi Medical University, Nagakute, Aichi, Japan

## Abstract

Stromal cells and osteoblasts play major roles in forming and modulating the bone marrow (BM) hematopoietic microenvironment. We have reported that FGF2 compromises stromal cell support of normal hematopoiesis. Here, we examined the effects of FGF2 on the leukemia microenvironment. *In vitro*, FGF2 significantly decreased the number of stromal-dependent and stromal-independent G0-leukemia cells in the stromal layers. Accordingly, CML cells placed on FGF2-treated stromal layers were more sensitive to imatinib. Conversely, FGF2 increased the proliferation of osteoblasts via FGFR1 IIIc, but its effects on osteoblast support of leukemia cell growth were limited. We next treated a human leukemia mouse model with Ara-C with/without systemic FGF2 administration. BM sections from FGF2-treated mice had thickened bone trabeculae and increased numbers of leukemia cells compared to controls. Leukemia cell density was increased, especially in the endosteal region in FGF2/Ara-C -treated mice compared to mice treated with Ara-C only. Interestingly, FGF2 did not promote leukemia cell survival in Ara-C treated spleen. Microarray analysis showed that FGF2 did not alter expression of many genes linked to hematopoiesis in osteoblasts, but modulated regulatory networks involved in angiogenesis and osteoblastic differentiation. These observations suggest that FGF2 promotes leukemia cell growth in the BM by modulating osteoblast functions.

Hematopoiesis, the formation of blood cells, is a dynamic process that includes self-renewal of hematopoietic stem cells (HSCs) in the bone marrow (BM), generation of lineage-committed cells, and mobilization of mature cells into the blood stream^1^. The BM niche is a microenvironment that anatomically harbors stem cells or progenitors and governs their fate^2^. The BM niche is comprised of many cell types, including predominantly BM mesenchymal stromal cells and their derivatives (especially osteoblasts)^3^,^4^. Interestingly, it has been reported that remodeling of the BM microenvironment is an indispensable event in the development of blood malignancies, and is involved in controlling the maintenance and activity of disease-initiating leukemia stem cells and their progeny^5^.

Fibroblast growth factor 2 (FGF2), and other structurally related polypeptides, are potent inducers of growth, survival, chemotaxis, and differentiation of a variety of cell types including neoplastic cells, and play key roles in morphogenesis, development, angiogenesis, bone formation, and wound healing^6^,^7^. Members of the FGF superfamily function by binding to heparan sulfate proteoglycans and FGF receptors (FGFRs)^8^. The FGFR superfamily consists of four members, designated FGFR1, FGFR2, FGFR3, and FGFR4. Alternative splicing of FGFR1, FGFR2, and FGFR3 transcripts increases the number of principal FGFRs to seven (FGFR1-IIIb, FGFR1-IIIc, FGFR2-IIIb, FGFR2-IIIc, FGFR3-IIIb, FGFR3-IIIc, and FGFR4)^8^. Elevated expression of FGF2 has been reported in hematological malignancies, and is prognostically significant for multiple myeloma, leukemia, myelodysplastic syndrome and malignant lymphoma^9^. Compelling studies revealed that FGF2 could facilitate hematological malignancies through autocrine proliferative effects, and paracrine functions such as neovascularization^9^. However, little is known about the effects of FGF2 on the BM microenvironment, even though both mesenchymal stromal cells and osteoblasts express FGFRs^10^,^11^.

To address this issue further, we evaluated mesenchymal stromal cell and osteoblast mediated support of leukemia cell growth after FGF2 exposure. We describe a novel role of FGF2 as a modulator of osteoblast and mesenchymal stromal cell function, and provide evidence for involvement of FGF2 in leukemia pathogenesis.

## Results

### FGF2 attenuates the leukemia supportive properties of stromal cells, but not osteoblasts

**Figure 1:**
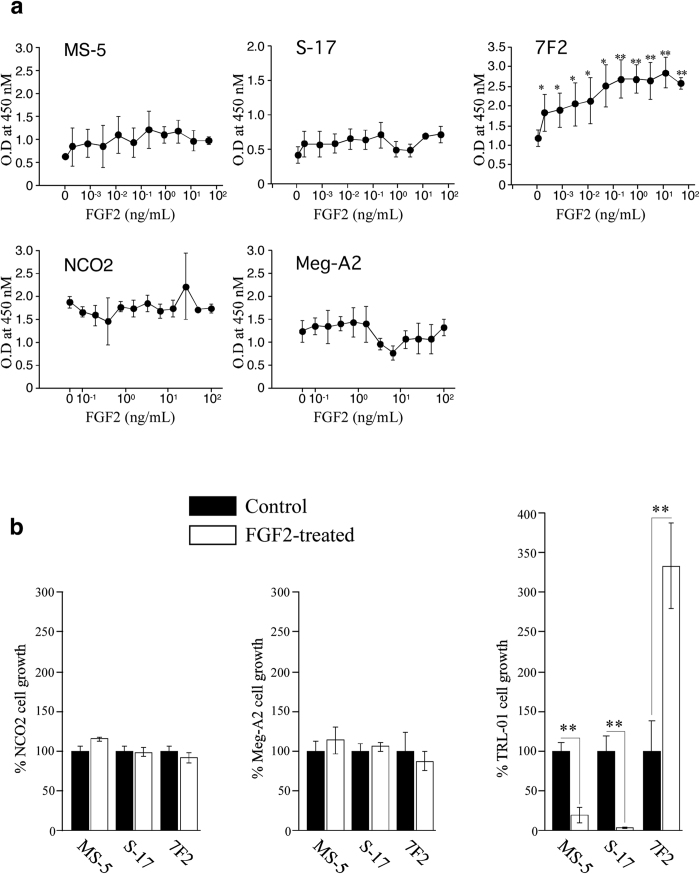
Effects of FGF2 on functions of stromal cells and osteoblasts. (**a**) The proliferation of mouse stromal cells (MS-5 and S-17), mouse osteoblasts (7F2), and human leukemia cells (NCO2 and Meg-A2) after exposure to FGF2 was evaluated by using a TetraColor One colorimetric assay. All experiments were performed in four independent sets and repeated twice. (**b**) The effects of FGF2-treated stromal or osteoblast layers on the proliferation of leukemia cell were evaluated by a short-term co-culture assay. MS-5, S-17 and 7F2 feeders (80~90% confluent) in 6-well plates were incubated with or without FGF2 (50 ng/ml) for 72 hours. After removal of supernatants, NCO2, Meg-A2 or TRL-01 cells (2.0 × 10^5^ in 2 mL of Iscove containing 10% FBS) were added in medium only, or with FGF2 (50 ng/mL). After 96-hour co-culture (triplicate cultures), non-adherent cells were removed, and viable cells were counted. The % growth of leukemia cells was calculated as (cell number with FGF2 or FGF2/cell number with no additive) × 100 (%). All experiments were repeated twice. The asterisk denotes statistical significance (**P < 0.01; *P < 0.05).

We first tested the effects of FGF2 on the proliferation of stromal cells (MS-5 and S-17), osteoblast cells (7F2), and leukemia cells (NCO2, Meg-A2 and TRL-01). FGF2 did not enhance the proliferation of MS-5 cells or S-17 cells significantly, consistent with previous data^10^, but proliferated 7F2 cells in a dose- dependent manner (Fig. 1a). Additionally, FGF2 uniformly induced morphological change in semi-confluent cultures of MS-5 and S-17 cells, which acquired a thinner and more elongated appearance, but 7F2 cells remained unchanged (data not shown). However, FGF2 had no effect on the proliferation of NCO2 or Meg-A2 cells (Fig. 1a). FGF-2 was not toxic and did not increase proliferation of TRL-01 cells cultured without supportive cells for 48 hours (data not shown. Later time points could not be examined because TRL-01 cells do not survive without supportive cells).

We next evaluated the effect of FGF2 treatment on the ability of MS-5, S-17 and 7F2 cells to support human TRL-01 stromal-dependent leukemia cells in culture. After a 96-hour incubation on MS-5, S-17 or 7F2 monolayers with or without FGF2, all non-adherent/loosely adherent cells were collected and viable cells were counted. The number of cells collected from the 7F2 monolayer with FGF2 was about three-fold greater than from the 7F2 monolayer without FGF2 (Fig. 1b, right panel). However, in the presence of FGF2, cell recovery from MS-5 and S-17 monolayers was significantly reduced (Fig. 1b, right panel), which could not be attributed to decreased stromal cell numbers, as treatment with FGF2 at 50 ng/mL for 96 hours did not affect stromal cell viability (Fig. 1a). However, similar effects of FGF2 on NCO2 and Meg-A2 human stromal-independent leukemia cells were not observed (Fig. 1b, left and middle panel).

### Effects of FGF2 on cell cycle and chemo-sensitivity of leukemia cells on stromal layers

**Figure 2:**
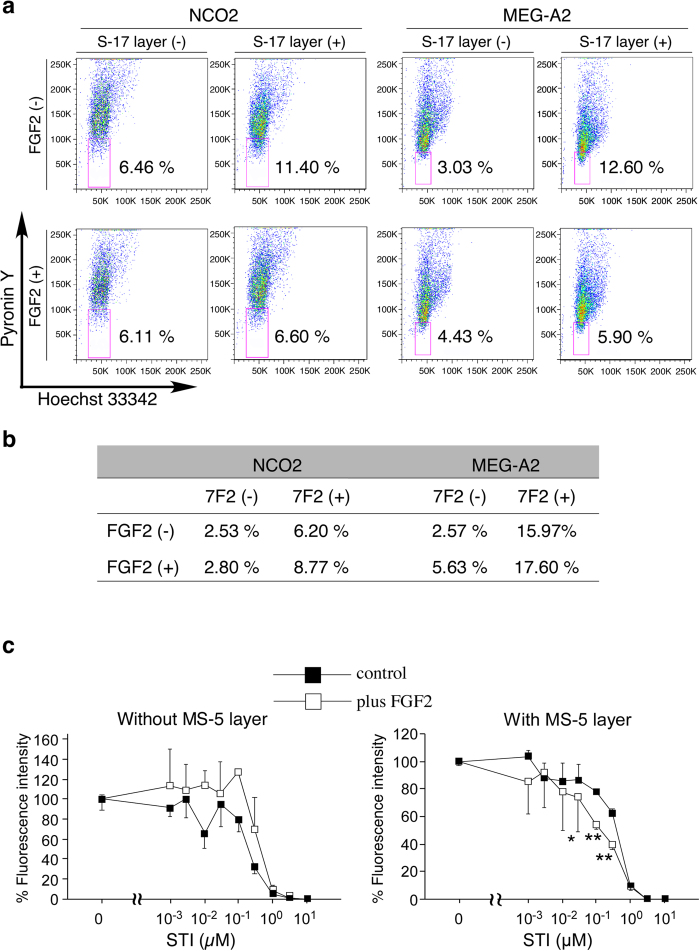
Evaluation of cell cycle and chemo-sensitivity of leukemia cells on FGF2-treated stromal or osteoblast layers. (**a**,**b**) Cell cycle of leukemia cells: NCO2 and Meg-A2 onto stromal cells: MS-5, S-17, or osteoblasts: 7F2 were analyzed by FACS with Hoechst and pyronin Y. MS-5, S-17 and 7F2 feeders (80~90% confluent) in 6-well plates were incubated with or without FGF2 (50 ng/ml) for 72 hours. After removal of supernatants, NCO2 or Meg-A2 (2.0 × 10^5^ in 2 mL of Iscove containing 10% FBS) were added in medium only, or with FGF2 (50 ng/mL). After 96-hour co-culture (triplicate cultures), non-adherent cells were harvested and resuspended in PBS containing 1 μg/ml Hoechst (Sigma) for 45 minutes at 37 °C, then 5 μl of 100 μg/ml pyronin Y (Sigma) was directly added to the cells. After 15-minute incubation, the cells were transferred onto ice. Hoechst was excited with the UV line of an Enterprise laser, and pyronin Y was excited with the 488 line of the same laser. All experiments were repeated twice. (**c**) Effects of FGF2 on the chemosensitivity of leukemia cell onto stromal layers were measured by using cells transduced with a fluorescent substance. NCO2; a cell lines derived from a CML patient, transfected with a GFP-expressing lentivirus vector, was seeded alone or on MS-5 feeder at a density of 5000 cells/well in 96-well plates. After 24 hours, a designated concentration of Imatinib mesylate (STI571) was added with or without 50 ng/ml FGF-2, and fluorescence intensity of each well was measured 3 days later by a microplate reader. All experiments were repeated twice. (**P < 0.01; *P < 0.05).

We found that FGF2 treatment attenuated the viability of human TRL-01 stromal-dependent leukemia cells as described above. We next evaluated the effects of FGF2 on human NCO2 and Meg-A2 stromal-independent leukemia cells, which did not require supportive cells to propagate. Cell cycle analysis showed that S-17 layers increased the fraction of both NCO2 and Meg-A2 in G_0_ phase (Fig. 2a) as well as MS-5 layers (NCO2 cells in the G_0_ phase without MS-5 layers: 6.18%, NCO2 cells in the G_0_ phase onto MS-5 layers: 12.1%), but the effect was perturbed by the addition of FGF2 (Fig. 2a, upper column). FGF2 alone (no stromal layers) did not alter the cell cycle of NCO2 or Meg-A2 cells (Fig. 2a). Interestingly, the osteoblast layer tended to increase the fraction of both NCO2 and Meg-A2 in G_0_ phase, which was not statistically significant though, but the effect was not perturbed by the addition of FGF2 (Fig. 2b). Since it was reported that quiescent leukemia cells were insensitive to chemotherapy^12^, we next treated NCO2 onto S17 cell (stromal) layers with imatinib mesylate (STI571) with or without FGF2. As shown in Fig. 2c, STI571 decreased the number of live NCO2 cells growing on S17 cell layers treated with or without FGF2 in a dose dependent manner. However, significantly fewer NCO2 cells were recovered from FGF2 and STI571-supplemented co-cultures than from co-cultures supplemented with STI571 alone. FGF2 (50 ng/mL) was not toxic for NCO2 cells as judged by fluorescence intensity at 0 ng/mL of STI571 and described above (Fig. 1a). These results clearly suggest that FGF2 enhanced the chemo-sensitivity of leukemia cells growing on stromal layers by inhibiting stromal functions to induce cell cycle arrest.

### Analysis of FGF receptor expression in osteoblasts

**Figure 3:**
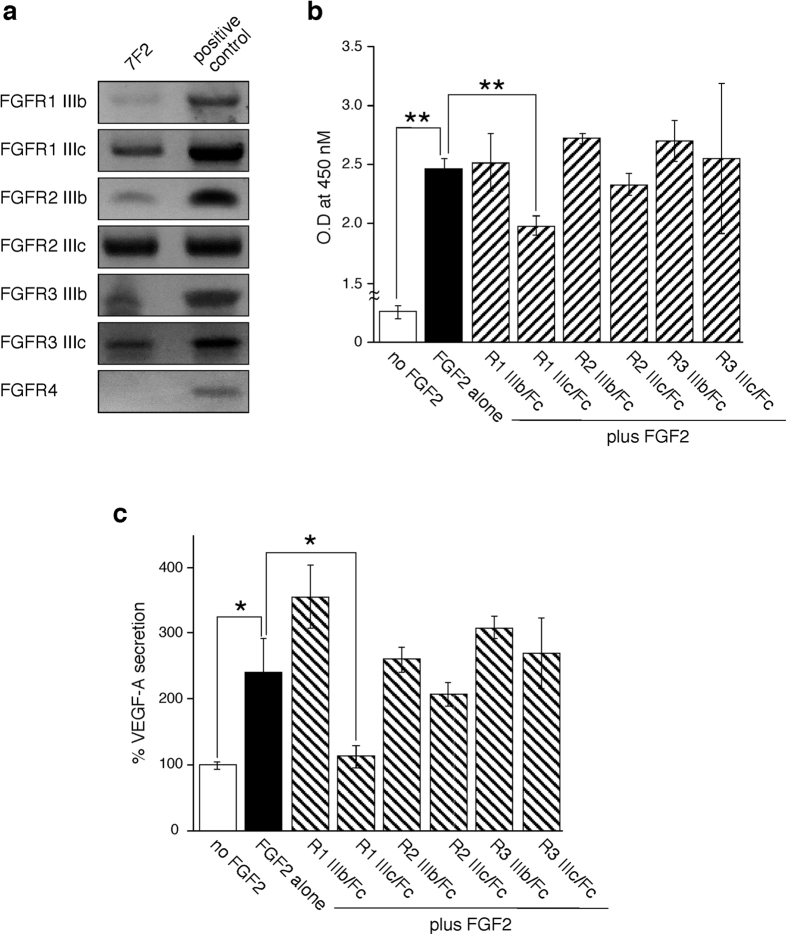
Expression of FGF receptors in osteoblasts. (**a**) Expression of FGFR transcripts in 7F2 cells was assessed by RT-PCR. Total RNA extracted from 7F2 cells was subjected to RT-PCR using specific primer pairs. PCR products were separated on 2% agarose gel prestained with 1 μg/ml ethidium bromide and visualized under UV light. After capturing images, extra parts of gels were cropped. Representative results from at least two independent experiments are shown. (**b**) FGFR subtype(s) responsible for FGF2-induced proliferation of osteoblasts was detected by an inhibition assay using FGFR/Fc chimeras. All experiments were performed in four independent sets and repeated twice. (**P < 0.01; *P < 0.05). (**c**) FGFR subtype(s) responsible for FGF2-induced VEGF-A secretion from osteoblasts was detected by a specific ELISA using FGFR/Fc chimeras. All experiments were performed in four independent sets and repeated twice. (**P < 0.01; *P < 0.05).

Since the FGFR expression profile of osteoblasts has not been unequivocally elucidated and remains controversial, we tested 7F2 cells for the presence of specific transcripts for each of the seven receptors. Using specific primer pairs and appropriate control mRNAs from mouse tissues (brain for* FGFR1 IIIb* and *FGFR1 IIIc*, lung for *FGFR2 IIIb, FGFR2 IIIc* and *FGFR3 IIIb*, liver for *FGFR4*) or cells (S-17 cells for *FGFR3 IIIc*)^10^, we detected expression of *FGFR1 IIIb, FGFR1 IIIc, FGFR2 IIIb, FGFR2 IIIc, FGFR3 IIIb* and *FGFR3 IIIc*, but not *FGFR4* (Fig. 3a).

### FGF2 facilitates osteoblast functions through FGFR1 IIIc

To identify which of these receptor subtypes is responsible for mediating FGF2-induced proliferation of osteoblasts, and VEGF-A secretion from osteoblasts, we used soluble Fc-fusion chimeras of the extracellular domain for each of the six candidate receptors (FGFR1 IIIb/Fc, FGFR1 IIIc/Fc, FGFR2 IIIb/Fc, FGFR2 IIIc/Fc, FGFR3 IIIb/Fc and FGFR3 IIIc/Fc). As expected, FGF2 (15 ng/mL) significantly enhanced the proliferation of 7F2 cells. Addition of FGFR1 IIIc/Fc chimera, but not FGFR1 IIIb/Fc, FGFR2 IIIb/Fc, FGFR2 IIIc/Fc, FGFR3 IIIb/Fc or FGFR3 IIIc/Fc, to FGF2-supplemented cultures attenuated the proliferation of 7F2 cells (Fig. 3b). In another setting, we measured the VEGF-A content in 72-hour culture by a specific ELISA. Control 7F2 cells with no additive secreted a large amount of VEGF-A (33.6 ng/mL in 200 mL supernatant per 10000 cells for 72 hours). As expected, FGF2 (15 ng/mL) increased significantly levels of VEGF-A 2.43 times more than controls. Addition of FGFR1 IIIc/Fc chimera to FGF2-supplemented cultures significantly decreased VEGF-A levels in culture supernatants, which were almost similar to those detected in supernatants of 7F2 cells cultured in medium alone, without FGF2 (Fig. 3c). By contrast, FGFR1 IIIb/Fc, FGFR3 IIIb/Fc, FGFR2 IIIb/Fc, FGFR2 IIIb/Fc, FGFR3 IIIb/Fc or FGFR3 IIIc/Fc did not inhibit VEGF-A levels (Fig. 3c). None of FGFR/Fc chimeras added alone to 7F2 cell cultures affected the growth of 7F2 cells or VEGF-A secretion from 7F2 (data not shown).

These results demonstrated that FGFR1 IIIc is the receptor that mediates FGF2-induced proliferation and VEGF-A secretion in osteoblasts.

### Effects of systemic FGF2 administration to mice bearing human primary leukemia cells

**Figure 4:**
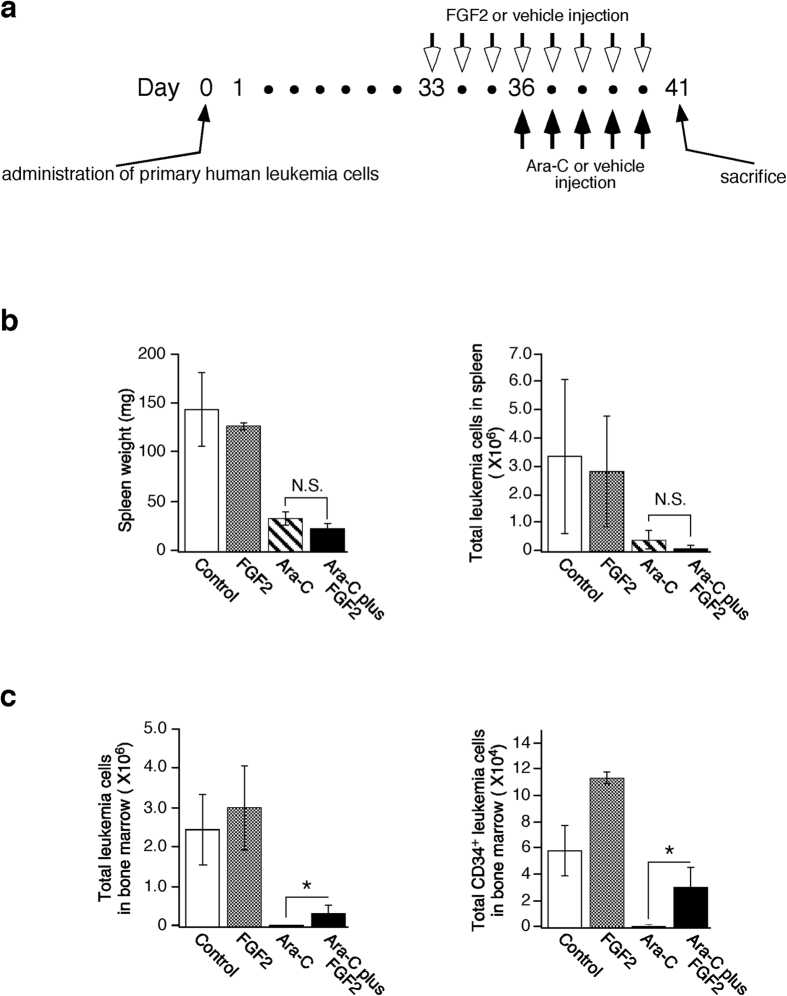
Evaluation of a human leukemia mouse model treated systemically with FGF2 plus/minus Ara-C. (**a**) Schematic representation of treatment regimen. A mouse human leukemia model was generated by engrafting non-obese diabetic/severe combined immunodeficient/interleukin (NOD/SCID/IL) 2rγ^null^ mice with primary leukemia cells as described elsewhere. Animal studies were repeated twice. (**b**,**c**) Evaluation of spleens and BM after addition of FGF2 with/without Ara-C treatment. After mice were sacrificed, spleens were removed and weighed (left panel). BM cells were flushed from femur and tibia with PBS. One femur was preserved for histological examination. Single-cell suspensions from the spleen and BM were used for cell counting and FACS analysis. (**P < 0.01; *P < 0.05).

**Figure 5:**
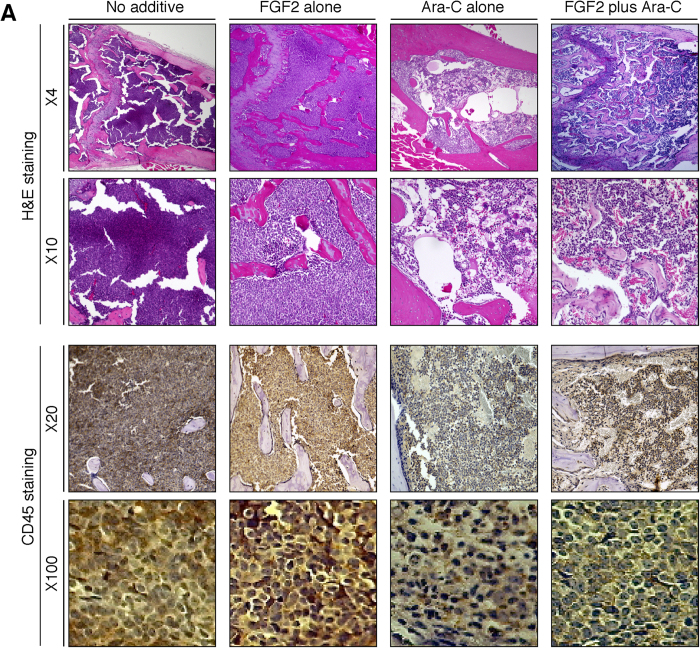
Representative microscopic images of BM from a human leukemia mouse model treated systemically with FGF2 plus/minus Ara-C. All mice bearing human leukemia cells were humanely killed five days after treatment with FGF2 with or without Ara-C, and one femur from each mouse was excised for histological evaluation. Sections of the femur were stained with H&E (top row ×4; 2nd row ×10) or immunostained for human CD45 (3rd row ×20; bottom row ×100), showing different degrees of leukemia cell infiltration and bone trabeculae.

As described above, *in vitro* analysis was discrepant, suggesting that FGF2 treatment decreased the supportive properties of stromal cells, while FGF2-treated osteoblasts were somewhat more supportive of leukemia cell growth. We next evaluated the effects of FGF2 on leukemia cells *in vivo* (Fig. 4a). Groups of six mice bearing human primary leukemia cells were pretreated daily with FGF2 injections i.v. (5 μg/mouse in 0.1 ml buffer) or buffer alone for three days, at which time each group was divided into two subgroups (three mice/subgroup). Mice pretreated with FGF2 received additional FGF2 for five days with Ara-C, or buffer alone. Similarly, mice not pretreated with FGF2 received Ara-C, or buffer alone. There was no evidence of toxicity during FGF2 treatment. After eight days of treatment, all mice were sacrificed. The spleens from mice bearing human leukemia cells were enlarged, and human leukemia cells were easily detectable (Fig. 4b). Both the spleen size and the number of leukemia cells in mice treated with Ara-C alone, and mice treated with Ara-C plus FGF2 treatment, were reduced compared to control mice. Interestingly, the spleen size and the number of leukemia cells in mice treated with Ara-C plus FGF2 tended to be lower compared to mice treated with Ara-C alone (Fig. 4b), which may be reflecting the *in vitro* results that FGF2 lowered the supportive properties of stromal cells toward leukemia cells. In BM, FGF2 treatment increased total numbers of leukemia cells including the number of CD34^+^ positive leukemia cells. Ara-C treatment significantly reduced total numbers of leukemia cells and CD34^+^ positive leukemia cells, which was partially alleviated by the addition of FGF2 (Fig. 4c). These results provide evidence that FGF2 can support the survival of leukemia cells in the bone marrow and not in the spleen. Histologically, BM sections from FGF2-treated mice and FGF2/Ara-C treated mice displayed thickened bone trabeculae, which was largely absent from the controls and the Ara-C treated mice (Fig. 5). The cell density within the marrow cavity in FGF2/Ara-C treated mice was higher than that from mice treated with Ara-C alone (Fig. 5, top and 2nd row), which was due to the increased number of leukemia cells (confirmed by CD45 staining, Fig. 5, 3rd and bottom row).

Other organs, including the kidneys, heart, lung and intestine from FGF2-treated mice appeared normal (data not shown). These facts suggest that the number of osteoblasts strongly correlated with the growth and survival of leukemia cells in the bone marrow.

### Analysis of FGF2 modulation of gene expression in osteoblasts

The results above suggested that FGF2 induces indirect effects from osteoblasts that are supportive of leukemia cell growth. Using microarray analysis, we screened 7F2 cells for genes regulated by FGF2 (48 hour culture with or without 50 ng/ml FGF2) (See Tables 1 and 2 for a selected subset of results). The entire list of genes significantly regulated by FGF2 can be found in Supplementary Table 1. FGF2 did not significantly alter expression of many genes previously linked to regulation of hematopoiesis, such as the *flt3-ligand, SCF*, or *G-CSF* (data not shown), but significantly modulated expression of genes related to angiogenesis such as *VEGF-A, Ang-1* and *CXCL12*. Also, addition of FGF2 significantly reduced expression of *Ibsp, Dlx5* and *Figf* that may be involved in osteoblastic differentiation^13^.

## Discussion

In hematological malignancies, elevated levels of FGF2 in the BM have been reported to have prognostic value^9^, although the reasons for this link remain unclear. Here, we provide direct evidence that FGF2 facilitates the growth and survival of leukemia cells in the BM, even though the direct effects of FGF2 on leukemia cell growth were minimal. In bone marrow, FGF2 treatment increased total numbers of leukemia cells, including CD34^+^ positive leukemia cells. Ara-C treatment significantly reduced total numbers of leukemia cells, including CD34^+^ positive cells, which was partially alleviated by the addition of FGF2 (Fig. 4c). CD34^+^ is a marker of leukemia stem cells associated with chemo-resistance and relapse^14^. An increase in the number of leukemia cells induced by FGF2 was coupled with the increased bone marrow endosteal region: osteoblasts (Fig. 5). Interestingly, the supportive effects of FGF2 on leukemia cell growth were not observed in the spleen (Fig. 4b), which does not contain osteoblasts. These results suggest that osteoblasts could play a pivotal role in regulating leukemia cell proliferation. However, recent evidence suggests that there is little direct association between osteoblasts and HSCs^15^,^16^ in accordance with *in vitro* results that FGF2-treated 7F2 cells showed limited supportive functions toward leukemia cells (Figs 1b and 2b), even though leukemic stem cells localize within the osteoblast-rich (endosteal) area of the BM where acute myeloid leukemia cells are protected from chemotherapy-induced apoptosis^17^. These paradoxical reports led to an increased interest in the vasculature adjacent to osteoblasts, composed of sinusoids with CXCL12- abundant reticular (CAR) cells^18^ and arterioles^16^, both of which are associated with HSCs. Importantly, arteriolar niches are known to maintain HSC quiescence^16^ and increased BM vascularity has been reported to closely correlate with disease progression in hematological malignancies^19^,^20^,^21^. Although the regulatory mechanisms involving the vasculature, especially arterioles, are poorly understood, osteoblast involvement is highly likely because 7F2 cells secrete a considerable amount of VEGF-A (described above) compared to other types of cells^22^, and the arteriolar niches exist in close proximity to osteoblasts^15^. The microarray data may provide clues about the mechanisms underlying FGF2 mediated support of the growth and survival of leukemia cells in the BM. Our data indicated that FGF2-stimulated 7F2 cells expressed more *VEGF-A* and, less *Ang-1and CXCL12* (*SDF-1*) than unstimulated 7F2 cells (Tables 1 and 2). VEGF-A is a potent proangiogenic factor that stimulates angiogenesis (primarily arterioles), and vascular permeability by interacting with the tyrosine kinase receptors VEGFR-1 and VEGFR-2^22^. It has been reported that VEGF-A and its receptor, VEGFR-2, could contribute to HSC regulation by promoting expansion of the HSC pool, supporting self-renewal, and preventing HSCs exhaustion^23^,^24^. Decreased Ang-1 could stimulate vascular sprouting, resulting in increased angiogenesis, because Ang-1 maintains and stabilizes vessels to inhibit angiogenesis under normal conditions^25^. Additionally, FGF2 can promote angiogenesis and induce long-lasting vascular networks synergistically with VEGF-A^26^. These data suggest that FGF2 could support the vascular niche by modulating osteoblast functions. However, little is known how CXCL12 regulates the vasculature in the bone marrow even though CXCL12 has been reported to exert angiogenic effects in several organs^27^,^28^,^29^. Further studies are required to elucidate the significance of FGF2-induced CXCL12 depletion in the bone marrow.

CXCL12 and its receptor, CXCR4, have emerged as critical regulators of hematopoiesis, mediating growth and survival of hematopoietic and myeloid progenitor cells and pre–B cells, and promoting the retention of immature blood cells in the bone marrow^30^. The CXCL12-CXCR4 axis plays an important role in the leukemia-microenvironment since CXCR4 inhibitors mobilize leukemia cells from bone marrow into circulation, and increase chemo-sensitivity^31^. We previously reported that FGF2 decreased CXCL12 expression in stromal cells and the total amount of CXCL12 in the bone marrow^32^. Concordantly, FGF2 lowered CXCL12 expression in osteoblasts as well (Table 2). The reason why decreased CXCL12 in the BM did not lead to a decreased number of leukemia cells is not known. TGF-β1 has been reported to enhance the retention of hematopoietic progenitor cells in the bone marrow even though TGF-β1 also reduced CXCL12 expression^33^. Itkin *et al*. has proposed that FGF2 could expand HSCs directly and promote stromal cell proliferation to compensate for FGF2-induced depletion of CXCL12^34^. Whether FGF2 influences the retention of hematopoietic progenitor cells in the BM remains to be determined. In our report, FGF2 had little proliferative effect on stromal cells and leukemia cells. Thus, the reasons why decreased CXCL12 expression in the BM did not lead to a decreased number of leukemia cell needs to be elucidated further.

FGFs exert their biological activities through the tyrosine kinase FGF receptors expressed on various cell types^35^. An essential feature of FGFRs is the existence of two alternative exons, IIIb and IIIc, which encode a different C-terminal portion of domain 3. We detected expression of FGFR1 IIIb, FGFR1 IIIc, FGFR2 IIIb, FGFR2 IIIc, FGFR3 IIIc and FGFR3 IIIc, but not FGFR4, in 7F2 cells (Fig. 3a). Importantly, mice with targeted disruption of the whole FGFR1 gene, or FGFR1 IIIc alone, died perinatally^36^,^37^, whereas mice with an inactive exon IIIb were viable and fertile, providing evidence that the FGFR1 IIIc isoform is responsible for most of the biological functions of FGFR1, and the IIIb isoform plays a minor role^38^. Consistent with FGFR1 IIIc playing a dominant role over FGFR1 IIIb, we found that FGFR1 IIIc is exclusively responsible for mediating FGF2-induced proliferation of 7F2 cells (Fig. 3b). Zhang *et al*. clearly showed that deletion of FGFR1 in osteoblasts resulted in increased CXCL12 expression in peripheral blood^39^ consistent with the microarray results. These data suggest important roles of FGFR1 in regulating osteoblast function, including proliferation and gene expression. We previously reported that FGF2 acted on stromal cells via FGFR1 IIIc, however the biological responses of stromal cells and osteoblasts are not always similar, even though osteoblasts are derived from mesenchymal stromal cells^40^. For instance, FGF2 induces differentiation of stromal cells into neural cells^41^, while it inhibits osteoblast maturation. It has been reported that FGF-FGFR signaling led to activation of three pathways: RAS-MAP kinase, P-I-3 kinase-AKT, and PLCG^35^. These facts suggest that the difference in biological response to FGF2 between stromal cells and osteoblasts depends on which pathways are utilized.

In conclusion, our data suggest that FGF2 may be a promising therapeutic target for hematological malignancies that propagate in the BM. Antibody neutralization and pharmacologic inhibition of activated tyrosine kinase receptors^42^ are effective treatment modalities that could be applied to FGF2 and its receptors.

## Materials and Methods

### Animal studies

The animal experiments were approved by the institutional ethics committee for Laboratory Animal Research, Nagoya University School of Medicine and Aichi Medical University, and were performed according to the guidelines of the Institutes.

### Reagents and Cells

Human recombinant FGF2 was from Peprotech Inc. (Rocky Hill, NJ, USA). Dulbecco’s modified Eagle’s medium (DMEM), Iscove’s modified Dulbecco’s medium (IMDM), heat-inactivated fetal bovine serum (FBS), minimal essential medium alpha (αMEM) and heat-inactivated horse serum were from Gibco-BRL (Invitrogen, Carlsbad, CA, USA). The mouse BM-derived stromal cell lines MS-5^43^ and S-17^44^ were obtained from Dr. A.C. Berardi (Ospedale Bambin Gesu, Rome, Italy) and Dr. K. Dorshkind (UCLA, Riverside, CA). The mouse osteoblast cell line, 7F2, was obtained from the American Type Culture Collection (Rockville, MD, USA). Stromal cell lines and 7F2 cells were maintained in αMEM containing 10% FBS. The profiles of the stroma-dependent human acute myeloid leukemia cell line TRL-01^45^, the human megakaryoblastic cell line MEG-A2^46^, and the chronic myeloid leukemia cell line NCO-2^47^ are described elsewhere.

### RNA preparation and reverse transcription-polymerase chain reaction analysis (RT-PCR) for FGFR expression in osteoblasts

Total RNA was extracted from 7F2 cells using TRIzol Reagent (Molecular Research Center, Cincinnati, OH, USA). RT-PCR to detect FGFR expression was carried out as described elsewhere^10^. RNA quality was evaluated in all samples by parallel RT-PCR for GAPDH. Absence of contaminating genomic DNA was ensured by RNA-PCR. PCR products were separated on a 2% agarose gel pre-stained with 1 μg/mL ethidium bromide and visualized under UV light.

### *In vitro* cell proliferation studies

The proliferative effects of FGF2 on stromal cells (MS-5 and S-17), mouse osteoblasts (7F2), and human leukemia cells (NCO2 and Meg-A2) were assessed by a colorimetric assay (TetraColor One; Seikagaku Co., Tokyo, Japan) as described elsewhere^48^. Briefly, cells were washed twice with PBS, suspended in culture medium (DMEM containing 10% FBS), plated (stromal cells: 1000 cells, osteoblast: 2000 cells, leukemia: 20000 cells/well in 0.2 mL culture medium) onto 96-well plates with the addition of FGF2 at various concentrations (0–100 ng/mL), and incubated for 72 hours. Subsequently, 10 μL of TetraColor One reagent was added to each well, and absorbance at 450 nm was measured eight hours later.

### *In vitro* inhibition assay using FGFR/Fc chimeras

FGFR Fc/chimeras (R&D Systems, Minneapolis, MN, USA) (1 μg/ml in αMEM containing 10% FBS) were pre-incubated with or without FGF2 (15 ng/mL) for 30 minutes, and then applied to osteoblast layers (~90% confluent). 7F2 cells were incubated in medium alone, FGF2 (15 ng/mL) alone, or FGF2 (15 ng/mL) plus each of the FGFR/Fc chimeras (1 μg/mL) for 72 hours, after which 10 μL of TetraColor One reagent was added to each well, and absorbance at 450 nm was measured eight hours later. In another setting, a specific ELISA (R&D) was used to measure VEGF-A in 72-hour culture supernatants. The % VEGF-A secretion was calculated as follows: (VEGF-A secretion in FGFR/Fc group/VEGF-A secretion in control group with no additive) × 100 (%). All experiments were performed at least in four independent sets and repeated twice.

### Short-term co-culture of human leukemia cells with MS-5, S-17 or 7F2 cells

The assay was performed as described^49^ with modification. MS-5, S-17 and 7F2 feeders (80~90% confluent) in 6-well plates were incubated with or without FGF2 (50 ng/ml) for 72 hours. After removal of supernatants, NCO2, Meg-A2 or TRL-01 cells (2.0 × 10^5^ in 2 mL of IMDM containing 10% FBS) were added in medium only, or with FGF2 (50 ng/mL). After 96-hour co-culture (triplicate cultures), non-adherent cells were removed, and viable cells were counted. The % growth of leukemia cells was calculated as (cell number with or without FGF2/cell number with no additive) × 100.

The effects of FGF2 on the chemo-sensitivity of leukemia cells on stromal layers were measured using cells expressing GFP. NCO2, a cell line derived from a CML patient in blast crisis, was transduced with a GFP-expressing lentivirus vector, and seeded onto an S-17 feeder layer at a density of 5000 cells/well in 96-well plates. After 24 hours, a designated concentration of Imatinib mesylate (STI571) was added with or without 50 ng/ml FGF2, and fluorescence intensity of each well was measured three days later on a microplate reader (Infinite M200, Tecan, Männedorf, Switzerland). All experiments were performed at least in triplicates and repeated twice.

### Mouse human leukemia model and Ara-C treatment with or without FGF2

The mouse human leukemia model was generated by engrafting non-obese diabetic/severe combined immunodeficient/interleukin (NOD/SCID/IL) 2rγ^null^ mice (Central Institute for Experimental Animals, Kawasaki, Japan) with primary leukemia cells as described elsewhere^50^. This study was approved by the institutional review board of Nagoya University Graduate School of Medicine and performed in accordance with the ethical guideline for clinical studies issued by Japanese government. Briefly, peripheral blood was obtained from a patient with acute myeloid leukemia after informed consent, and mononuclear cells were isolated by Ficoll-Hypaque density centrifugation. Thirty days after intravenous injection of the mononuclear cells into the mice, the engraftment of human leukemia cells was confirmed by FACS with an anti-human CD45 antibody. The mice bearing human leukemia cells received Ara-C treatment with/without FGF2 as described below. Groups of six mice were pretreated with 100 μl of vehicle (PBS) intravenously (i.v.) *via* the tail vein daily for three days between days 33 and 35 post-inoculation with leukemia cells, and were divided into two subgroups of three mice each. Each subgroup received Ara-C (200 mg/kg in 100 μL of PBS) or vehicle (100 μL of PBS) for five days (Fig. 4a). Similarly, groups of six mice were pretreated with 5 μg of human recombinant FGF2 in 100 μl PBS (six mice) i.v. *via* the tail vein daily for three days. As above, six mice were divided into two subgroups (three mice each), and each subgroup received Ara-C alone (200 mg/kg in 100 μL of PBS) or Ara-C (200 mg/kg/day) with FGF2 (5 μg/body/day) for five days (Fig. 4a). All mice were sacrificed on day 41. Spleens were removed, weighed, and dissociated by mincing with fine scissors. To evaluate leukemia cell numbers in the BM, the ends of the femur and tibia were removed, and BM was flushed and passed through a nylon mesh to remove small pieces of bone and debris. One femur was preserved for histological examination. Single-cell suspensions from the spleen and the bone marrow were used for FACS analysis. Animal studies were repeated twice.

### Flow cytometry

Cells were stained with fluorescein-labeled murine monoclonal antibodies to human CD34, CD45, or with fluorescein-labeled isotype-matched antibodies (2 μg/mL at 4 °C) for 45 minutes (all antibodies from Becton Dickinson). Results from 1.0 × 10^4^ viable cells were collected using a FACScalibur cytofluorometer (Becton Dickinson) and analyzed using CELLQuest software (Becton Dickinson).

For cell cycle analysis, cells were labeled simultaneously by Hoechst and pyronin Y as described elsewhere^51^. Briefly, cells (~10^6^ cells/ml) were harvested and resuspended in PBS containing 1 μg/ml Hoechst (Sigma) for 45 minutes at 37 °C, then 5 μl of 100 μg/ml pyronin Y (Sigma) was directly added to the cells. After a 15-minute incubation, the cells were transferred onto ice. Hoechst was excited with UV using an Enterprise laser, and pyronin Y was excited at 488 nm using the same laser. Data were collected and analyzed as described above.

### Microarray analysis

7F2 cells were incubated for 48 hours in medium alone or with 50 ng/mL of FGF2 (duplicate cultures), and total RNA extracted with TRIzol Reagent. After RNA isolation, all the subsequent technical procedures including quality control and concentration measurement of RNA, cDNA synthesis and biotin-labeling of cRNA, hybridization onto mouse genome 430 2.0 oligonucleotide arrays which contain probes for detecting ~39000 transcripts (Affymetrix, Santa Clara, CA) and scanning of the arrays were carried out by Kurabo Biomedical Business (Osaka, Japan) according to the manufacture’s protocol. After background subtraction, low-quality measurements were excluded from further analysis and treated as missing values. Average intensities for each spot in the FGF2-treated hybridization were divided by the average intensity in the untreated hybridization. Ratios were normalized based on the distribution of all targets on the array. The calibrated ratios from the duplicate set were averaged.

### Statistical analysis

The statistical significance of group differences was evaluated using the Student’s t-test between two groups and ANOVA followed by Mann-Whitney’s test for multiple comparisons using SPSS software (IBM Inc, Armonk, NY, USA). Statistical differences between groups were considered significant at *P* < 0.05.

## Additional Information

**How to cite this article**: Sugimoto, K. *et al*. Fibroblast Growth Factor-2 facilitates the growth and chemo-resistance of leukemia cells in the bone marrow by modulating osteoblast functions. *Sci. Rep.* **6**, 30779; doi: 10.1038/srep30779 (2016).

## Figures and Tables

**Table 1:** Profiling of highly up-regulated genes after FGF2 exposure.

Gene Symbol	UniGene ID	Control average	FGF2 average	log2 ratio	Gene function
Aldh18a1	Mm.233117	423.4	1059.33	1.323	proline biosynthetic process
Igfbp4	Mm.233799	199.89	502.91	1.331	regulation of cell growth
Slc3a2	Mm.4114	737.44	1855.8	1.331	carbohydrate metabolic process
Pla2g4a	Mm.4186	173.5	440.59	1.344	ovulation from ovarian follicle
Oxnad1	Mm.202257	73.03	187.05	1.357	oxidation-reduction process
Efnb2	Mm.209813	122.81	327.22	1.414	angiogenesis
Hk2	Mm.255848	99.06	265.49	1.422	carbohydrate metabolic process
Fxyd5	Mm.1870	98.63	266.32	1.433	ion transport
Pck2	Mm.29856	437.49	1192.43	1.447	gluconeogenesis
Mtbp	Mm.390829	109.51	300.02	1.454	cell cycle arrest
Sox9	Mm.286407	150.94	419.28	1.474	skeletal system development
Adssl1	Mm.3440	137.24	381.46,	1.475	purine nucleotide metabolic process
Slc30a4	Mm.27801	442.23	1233.74	1.48	ion transport
Pdia4	Mm.2442	215.3	612.85	1.509	glycerol ether metabolic process
Tnfaip2	Mm.255332	121	350.83	1.536	angiogenesis
Shmt2	Mm.29890	646.21	1874.86	1.537	glycine metabolic process
Asns	Mm.2942	390.33	1150.26	1.559	asparagine biosynthetic process
Psph	Mm.271784	117.81	350.07	1.571	cellular amino acid biosynthetic process
Itga6	Mm.225096	101.61	312.63	1.621	cell adhesion
Ptgs1	Mm.275434	110.81	344.85	1.638	prostaglandin biosynthetic process
Vegfa	Mm.282184	581.59	1844.79	1.665	angiogenesis
Glce	Mm.24411	93.92	306.52	1.707	glycosaminoglycan biosynthetic process
Wars	Mm.38433	278.19	942.01	1.76	angiogenesis
Slc6a9	Mm.244549	69.4	235.35	1.762	amino acid transmembrane transport
Gnpnat1	Mm.312945	262.23	894.46	1.77	glucosamine metabolic process
Slc7a5	Mm.27943	536.41	1834.84	1.774	amino acid transmembrane transport
Rps6ka2	Mm.268383	211.14	726.49	1.783	mitotic metaphase
Mthfd2	Mm.443	280.94	967.4	1.784	one-carbon metabolic process
Got1	Mm.19039	297.57	1072.98	1.85	oxaloacetate metabolic process
Hyou1	Mm.116721	356.72	1327.73	1.896	response to hypoxia
Slc7a1	Mm.275489	85.44	321.68	1.913	amino acid transmembrane transport
Slc20a1	Mm.272675	133.78	524.25	1.97	ion transport
Cyb5r1	Mm.280230	93.62	368.25	1.976	steroid biosynthetic process
Hspa5	Mm.330160	423.59	1671.18	1.98	anti-apoptosis
Steap1	Mm.85429	113.69	452.85	1.994	ion transport
Aldh3a1	Mm.4257	354.18	1438.4	2.022	cellular aldehyde metabolic process
Sesn2	Mm.23608	80.94	344.09	2.088	cell cycle arrest
Aldh1l2	Mm.263138	117.96	513.09	2.121	one-carbon metabolic process
Gpt2	Mm.200423	162.21	821.3	2.34	2-oxoglutarate metabolic process
Chka	Mm.225505	53.81	283.2	2.396	phosphatidylethanolamine biosynthetic process
Sema7a	Mm.335187	58.44	359.47	2.621	inflammatory response
Prl2c2	Mm.88796	203.29	1292.84	2.669	sprouting angiogenesis
Sdf2l1	Mm.30222	188.2	1213.98	2.689	endoplasmic reticulum-stress inducible-gene
Ptgs2	Mm.292547	166.34	1287.09	2.952	prostaglandin biosynthetic process
Atf3	Mm.2706	61.28	546.52	3.157	gluconeogenesis
Chac1	Mm.35083	73.02	659.22	3.174	apoptotic process

**Table 2:** Profiling of highly down-regulated genes after FGF2 exposure.

Gene Symbol	UniGene ID	Control average	FGF2 average	log2 ratio	Gene function
Abi3bp	Mm.209236	813.7	52.31	−3.959	positive regulation of cell-substrate adhesion
Postn	Mm.236067	506.26	33.26	−3.928	cell adhesion
Ibsp	Mm.4987	178.44	12.05	−3.889	ossification
Adh1	Mm.2409	266.52	26.19	−3.347	retinoid metabolic process
Dlx5	Mm.4873	268.37	32.93	−3.027	ossification
Hp	Mm.26730	665.68	83.45	−2.996	liver development
Cxcl12	Mm.303231	2209.16	277.5	−2.993	patterning of blood vessels
Col12a1	Mm.3819	818.76	108.71	−2.913	cell adhesion
Fat4	Mm.316210	111.61	18.54	−2.59	cell adhesion
Itga11	Mm.34883	100.42	20.56	−2.288	cell adhesion
Sfrp1	Mm.281691	1542.2	326.86	−2.238	somitogenesis/hemopoietic progenitor cell differentiation
Mest	Mm.335639	103.49	25.01	−2.049	regulation of lipid storage
Lfng	Mm.12834	235.02	57.53	−2.031	ovarian follicle development
Ccdc80	Mm.181074	437.18	108.21	−2.014	positive regulation of cell-substrate adhesion
Gas6	Mm.3982	261.7	66.55	−1.975	neuron migration
Cnn1	Mm.4356	404.24	104.97	−1.945	actomyosin structure organization
Figf	Mm.297978	606.69	160.65	−1.917	angiogenesis
Angpt1	Mm.309336	405.1	114.19	−1.827	angiogenesis
Lpl	Mm.1514	1375.74	394.29	−1.803	lipid metabolic process
Fbln5	Mm.288381	203.32	59.49	−1.773	cell adhesion
Sepp1	Mm.392203	146.26	43.57	−1.747	selenium compound metabolic process
Alpl	Mm.288186	1135.37	345.37	−1.717	endochondral ossification
P4ha3	Mm.150294	213.41	67.82	−1.654	oxidation-reduction process
Tagln	Mm.283283	633.62	207.79	−1.608	cytoskeleton organization
Enpp2	Mm.250256	1002.61	353.47	−1.504	chemotaxis
Lmo7	Mm.486662	197.58	71.84	−1.46	cell adhesion
Lox	Mm.172	2630.61	958.32	−1.457	blood vessel development
Acta2	Mm.213025	5827.37	2135.44	−1.448	regulation of blood pressure
